# Relationship of redundant Th17 cells and IL-17A, but not IL-17 F, with the severity of obstructive sleep apnoea/hypopnoea syndrome (OSAHS)

**DOI:** 10.1186/1471-2466-14-84

**Published:** 2014-05-15

**Authors:** Lin Ying, Hequan Li, Zhijie Pan, Shanni Ma, Pei Zhang, Qing Wang, Guohua Lu, Jianying Zhou

**Affiliations:** 1 Department of Respiratory Diseases, First Affiliated Hospital of Zhejiang, University School of Medicine, Zhejiang University, Zhejiang, P.R. China

**Keywords:** Obstructive sleep apnea hypopnea syndrome, Th17 cell, IL-17, Pulmonary arterial hypertension, Inflammation

## Abstract

**Background:**

The pathogenesis of obstructive sleep apnoea/hypopnoea syndrome (OSAHS), a highly prevalent disease, is not completely understood. The purpose of this study was to investigate the contributions of Th17 cells and the Th17-associated cytokines IL-17A and IL-17 F to OSAHS.

**Methods:**

46 male patients with a clinical suspicion of OSAHS were enrolled and divided into four groups based on their polysomnography results: controls and mild, moderate, and severe OSAHS. The serum levels of IL-17A and IL-17 F were determined by enzyme linked immunosorbent assay (ELISA), pulmonary arterial pressure (PAP) was determined by echocardiography, and Th17 cell frequencies in peripheral blood were measured by flow cytometry.

**Results:**

Serum IL-17A levels in the severe group were elevated (median value: control group 0.89 pg/ml, mild OSAHS 1.02 pg/ml, moderate OSAHS 1.18 pg/ml, and severe OSAHS 1.62 pg/ml; p < 0.05) and positively correlated with AHI (r = 0.52, p < 0.05) but negatively related to the mean O_2_ saturation and lowest O_2_ saturation (r = −0.349, p < 0.05; and r = −0.336, p < 0.05, respectively). Although the frequencies of Th17 cells in the OSAHS groups were higher than that in the control group, these differences were not significant (p = 0.275). Pulmonary arterial hypertension was not present in our patients as the median PAP of the normal control and the mild, moderate, and severe OSAHS groups were 26, 27.5, 24.5, and 25.5 mmHg, respectively (p = 0.676).

**Conclusion:**

IL-17A may be involved in the pathogenesis of OSAHS and may represent a target for therapeutic intervention.

## Background

Obstructive sleep apnoea/hypopnoea syndrome (OSAHS) is a breathing disorder that occurs during sleep. The prevalence of OSAHS among adults is high, with estimates of 3-7% of men and 2-5% of women [1]. The main characteristics of OSAHS are repeated episodes of partial or complete obstruction of the upper airway, which causes markedly reduced (hypopnoea) or absent (apnoea) airflow, and intermittent hypoxia and sleep deprivation that result from these episodes of obstructed breathing. OSAHS has been shown to be an independent risk factor for cardiovascular and cerebrovascular diseases such as systemic arterial hypertension, coronary artery disease, heart failure, and stroke [2,3]. The mechanisms involved in the associations between OSAHS and vascular diseases are also complex. Animal and clinical data suggest that intermittent hypoxia in OSAHS impairs endothelial function by altering the regulation of endothelial vasomotor tone and repair capacity while promoting vascular inflammation and oxidative stress [4].

Many immune system cell types, particularly T lymphocytes, participate in the pathogenesis of OSAHS. Dyugovskaya and colleagues reported that the CD8^+^ T lymphocytes from obstructive sleep apnoea patients undergo both phenotypic and functional changes, rendering them cytotoxic to target cells such as vascular endothelial cells [5]. Recently, Tan et al. examined the populations of Th1, Th2, and Treg cells in the peripheral blood of children with OSAHS and demonstrated that paediatric OSA is associated with a reduced Treg population and a Th1/Th2 balance that is shifted toward Th1 predominance [6].

Th17 cells, a recently discovered subset of T helper cells with a highly inflammatory nature, can secrete several potent proinflammatory cytokines, including IL-17A and IL-17 F, and are broadly implicated in inflammatory and autoimmune diseases [7-9]. However, the pathological effects of Th17 cells and their associated cytokines on OSAHS have not been determined. Kobi Sade and colleagues demonstrated that decreased ratios of Th17/Treg subpopulations may be involved in the pathogenesis of adenoid hypertrophy [10], which is the main cause of paediatric OSAHS [11]. In this study, we have explored the contributions of Th17 cells and their associated cytokines, IL-17A and IL-17 F, to OSAHS in adults.

## Methods

### Patients

Among the patients who visited our sleep disorders centre with a clinical suspicion of OSAHS, 46 male patients were enrolled in the study. All subjects underwent full-night polysomnography (Artisan, Rembrandt, USA), and 37 patients were diagnosed as having OSAHS, with an apnoea-hypopnoea index (AHI) > 5 (mild:AHI 5 ~ 15, n = 10; moderate: AHI 15 ~ 30, n = 11; severe: AHI > 30, n = 16). The AHI is the average number of apnoea and hypopnoea episodes per hour of sleep. No patient had previously been diagnosed with or treated for OSAHS. All subjects were free of any acute or chronic infection, cardiovascular diseases, and cancer and were taking no medications. This study was approved by the Ethics Committee of the First Affiliated Hospital of the Zhejiang University School of Medicine, Zhejiang University. Written informed consent was obtained from all subjects.

### Polysomnography

The presence and severity of OSAHS were determined by standard overnight polysomnography using electroencephalogram (EEG), electrooculogram (EOG), electrocardiogram (ECG), chin electromyogram, and measurements of oxygen saturation, airflow, and costal and abdominal movements when breathing. Apnoea was defined as a ≥90% decrease in airflow for at least 10 s relative to baseline. Hypopnoea was defined as a ≥50% decrease in airflow relative to baseline, with an associated ≥3% oxygen desaturation lasting at least 10 s [12]. As measured by AHI, there were three levels of OSAHS: mild (5 ≤ AHI < 15), moderate (15 ≤ AHI < 30), and severe (AHI ≥ 30).

### Measurement of IL-17A and IL-17 F levels

Blood samples were obtained from the antecubital vein on the morning after polysomnography and then centrifuged immediately at 3,000 rpm for 10 min. Plasma samples were stored at −80°C until use. The IL-17A and IL-17 F levels were determined quantitatively using a platinum enzyme-linked immunosorbent assay (eBioscience, San Diego, CA). The minimum detectable doses of IL-17A and IL-17 F were 0.23 pg/ml and 31.3 pg/ml, respectively, and the intra-assay coefficient of variation was <10%.

### Flow cytometry

Because we found a correlation between the serum level of IL-17 and OSAHS, we conducted follow-up visits with the study subjects to conduct additional research. However, only 17 subjects received a follow-up appointment and participated further in our research.

Intracellular cytokines were studied by flow cytometry. FITC-labelled anti-human CD4 (eBioscience) and PE-labelled anti-human IL-17 (eBioscience) were used to detect the Th17 cells. PBMCs, obtained from heparinised peripheral whole blood (400 μl), were added to 1 ml of 1640 medium (PAA Laboratories, Pasching, Austria) and incubated for 6 h at 37°C with 5% CO_2_ in the presence of 50 ng/mL phorbol myristate acetate (PMA) (BioVision, Mountain View, CA), 500 ng/mL ionomycin (Fermentek, Jerusalem, Israel), and 1 μl of GolgiPlug (eBioscience). The cells were then stained with FITC-labelled anti-human CD4. After fixation and permeabilisation, the cells were stained with PE-labelled anti-human IL-17. Finally, the stained cells were analysed by flow cytometry using a FACS Calibur (BD Biosciences), and the results were analysed with CellQuest software (BD Biosciences).

### Measurement of pulmonary arterial pressure (PAP)

Doppler echocardiography, using Vivid 7 (GE Vingmed Ultrasound, Horten, Norway) with a 2.5-MHz transducer (Accuson Cypress, Siemens, Germany), was performed by an experienced sonographer blinded to the study. The peak velocity of the tricuspid regurgitation jet was used to assess the PAP.

### Statistical analysis

Statistical analysis was performed using SPSS statistical software (SPSS, version 16.0 for Windows; SPSS Inc., Chicago, IL). The data were analysed using the Kruskal-Wallis and Mann–Whitney tests. The Pearson test was used to assess the correlation between variables. Differences were considered significant at p < 0.05.

## Results

### Demographic and sleep characteristics of subjects

A total of 46 male patients were analysed, with 37 in the OSAHS group (AHI ≥ 5) and 9 in the control group (AHI < 5). The characteristics of the study population are shown in Table 1. The median ages of the normal control and the mild, moderate, and severe OSAHS groups were 50, 47.5, 42, and 43.5 years, respectively (p = 0.823). The median BMI was 26.9, 26,and 29.4 kg/m^2^ in the three OSAHS groups, respectively, and 24.6 kg/m^2^ in the control group (p = 0.029). In the severe group, the median oxygen saturation (MSaO_2_) and lowest arterial oxygen saturation (LSaO_2_) were significantly decreased compared with the mild and moderate groups.

**Table 1:** Demographic and polysomnographic characteristics of the subjects (n = 46)

	**Control group (n = 9)**	**Mild OSAHS (n = 10)**	**Moderate OSAHS (n = 11)**	**Severe OSAHS (n = 16)**	**p value**
Age (years)	50(22,63)	47.5(39,61)	42(30,65)	43.5(25,70)	0.823
BMI (kg/m^2^)	24.6(22,30.8)	26.9(22.6,30.8)	26(22.5,31.6)	29.4(23.7,38.4)*	0.029
AHI (events/h)	1.2(0.1,3.6)	8.6(5.7,15)*	26.2(15.1,30)*#	50.7(40.7,91.4)*△	0.000
Lowest SaO_2_ (%)	91(86,95)	81(67,88)*	80(69,87)*	63(32,79)*#△	<0.001
Mean SaO_2_ (%)	97(96,98)	96(94,97)	96(94,98)	90.5(85,96)*#△	<0.001

The Kruskal-Wallis test was used to assess the differences among groups, and the Mann–Whitney test was used to test the significance of the differences between two groups.

The data were summarised as median (minimum,maximum). * p < 0.05 versus control group; # p < 0.05 versus mild OSAHS group; △ p < 0.05 versus moderate OSAHS group.

### Elevated level of serum IL-17A is observed in patients with OSAHS

**Figure 1:**
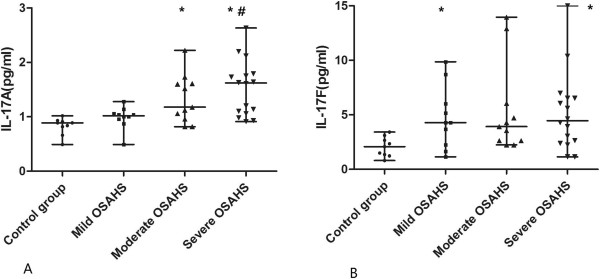
**Serum level of IL-17A and IL-17 F were assessed by ELISA.** The data are presented as median (minimum, maximum). * p < 0.05 versus control group; # p < 0.05 versus mild OSAHS group.

The concentrations of IL-17A and IL-17 F were determined by ELISA in our experiments. The median IL-17A level for normal controls was 0.8 pg/ml, which was lower than that for the OSAHS group. Moreover, the patients with more severe OSAHS tended to have higher IL-17A levels; this difference was significant (p < 0.001) (Figure 1A).

The serum level of IL-17 F in the OSAHS group was higher than that in the normal group (p < 0.05). However, the differences among the OSAHS groups were not significant (p > 0.05) (Figure 1B).

### The concentration of IL-17A is related to the severity of OSAHS

**Figure 2:**
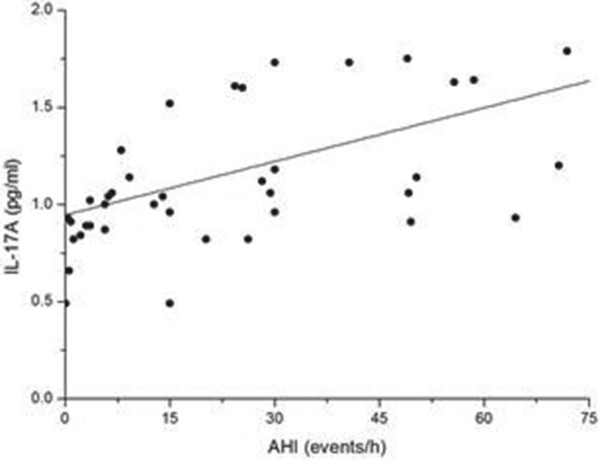
**The relationship between AHI and IL-17A level.** There was a significant positive relationship between AHI and the IL-17A level (r = 0.520, p < 0.01).

**Figure 3:**
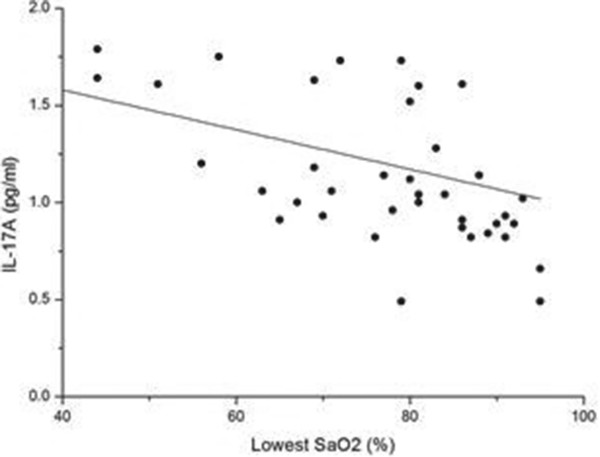
**The relationship between LSaO**_**2 **_**and IL-17A level.** There was a significant negative relationship between LSaO_2_ and the IL-17A level (r = −0.336, p < 0.05).

**Figure 4:**
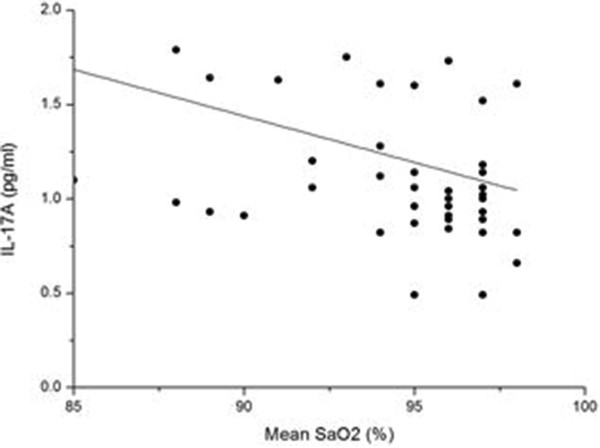
**The relationship between MSaO**_**2 **_**and the IL-17A level.** There was a significant negative relationship between MSaO_2_ and IL-17A level (r = −0.349, p < 0.05).

We found that the serum level of IL-17A in the OSAHS groups was higher than that in the normal control group. Further regression analysis showed that the concentration of IL-17A was positively correlated with AHI (r = 0.520, p < 0.01) (Figure 2) and negatively correlated with LSaO_2_ (r = −0.336, p < 0.05) (Figure 3) and MSaO_2_ (r = −0.349, p < 0.05) (Figure 4).

### The frequency of Th17 cells in peripheral blood has an elevated trend in the OSAHS group

**Figure 5:**
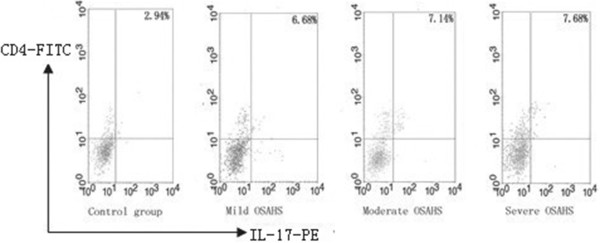
**The percentage of CD4**^**+**^**IL-17**^**+ **^**cells.** Single-cell suspensions prepared from the peripheral blood of 17 patients were double-stained with FITC-conjugated anti-CD4 and PE-conjugated anti-IL-17 and were analysed by flow cytometry.

Among 46 patients, 17 patients did not receive any treatment after diagnosis and were in the process of having follow-up appointments. Table 2 lists each parameter. The percentages of peripheral blood CD4^+^T lymphocytes that were also positive for intracellular IL-17 (i.e., Th17/CD4^+^ T cells) were determined for these patients (Figure 5). The frequencies of Th17 cells in the OSAHS groups trended higher compared with that in the control group; however, the frequencies decreased with increasing OSAHS severity. Overall, these differences were not significant (p = 0.275).

**Table 2:** Demographic and polysomnographic parameters, IL-17A concentrations, Th17 cell percentages, and PAP of the subjects (n = 17)

	**Control group (n = 3)**	**Mild OSAHS (n = 4)**	**Moderate OSAHS (n = 4)**	**Severe OSAHS (n = 6)**	**p value**
Age (years)	50(37,61)	46(39,59)	47.5(35,65)	50.5(42,59)	0.979
BMI (kg/m^2^)	26.1(23.8,30.8)	26.1(24.8,30.4)	25.5(22.5,31.1)	27.3(23.7,35)	0.787
Lowest SaO_2_ (%)	89(86,90)	75(67,84)	83(72,87)	65(44,79)	0.013
Mean SaO_2_ (%)	96(96,96)	96.5(95,97)	96.5(94,98)	92(88,96)	0.055
AHI (events/h)	2.2(0.8,3.6)	6.5(5.7,15)	26.3(23.4,30)	60.8(40.7,91.4)	0.002
IL-17A (pg/ml)	0.07(0.02,0.14)	0.09(0.03,0.11)	0.1(0.01,0.31)	0.37(0.08,1.36)	0.065
Th17%	2.2(0.6,6.0)	8.5(4.3,9.5)	6.6(2.7,13.4)	6.6(4,10.6)	0.275
PAP (mmHg)	26(23,26)	27.5(23,30)	24.5(23,26)	25.5(22,30.6)	0.676

### PAH was not present in our patients

A resting mean pulmonary artery pressure (PAPm) >25mmHg is the cut-off value for the diagnosis of manifest pulmonary arterial hypertension (PAH), as set forth at the 4th World Symposium on Pulmonary Hypertension held at Dana Point in the United States in 2008 [13]. In our study, the PAP was calculated by echocardiography in 17 patients. Our data did not support a diagnosis of PAH in our patients as the median PAP of the normal control and the mild, moderate, and severe OSAHS groups was 26,27.5,24.5,and 25.5 mmHg, respectively (p = 0.676) (Table 2).

## Discussion

Redundant Th17 cells and their associated cytokines have been shown to play a pathogenic role in autoimmune diseases and chronic inflammatory diseases, and these cells and cytokines may mediate many pathophysiological processes that occur with hypoxia. To investigate the pathogenic role of Th17 cells and IL-17 in intermittent hypoxia, we measured the serum levels of IL-17A and IL-17 F, PAP, and circulating Th17 cell frequencies in patients with OSAHS.

OSAHS is a common chronic sleep disorder, the pathogenesis of which is uncertain and likely to be a multifactorial process that includes sympathetic excitation, inflammation, and oxidative stress. Inflammation has been confirmed to play a crucial role due to the occurrence of recurrent hypoxia [14]. The unique form of hypoxia present in OSAHS, with repetitive short cycles of desaturation followed by rapid reoxygenation, termed intermittent hypoxia, similar to ischemia-reperfusion injury, is associated with a series of inflammatory responses through the induction of adhesion molecules and CRP in vascular endothelial cells and leukocytes [15,16]. Numerous studies have described an increase in the levels of systemic biomarkers of inflammation, including CRP and IL-6, in patients with OSAHS [17-19].

IL-6 is known to be an essential cytokine involved in inducing the differentiation of Th17 cells in both humans and mice. In the presence TGF-β, IL-6 activates the expression of the critical Th17 transcription factor RORC (RORγt in mice) through the Jak/STAT3 pathway during Th17 cell development [20,21]. In view of this role of IL-6, we hypothesised that IL-17 levels may be elevated in patients with OSAHS, and our results confirm this hypothesis.

Our study made several novel observations. First, the serum levels of IL-17A, a proinflammatory cytokine, are increased in patients with OSAHS; second, these elevated levels of IL-17A are positively correlated with the severity of OSAHS. Although there were significant differences in the IL-17A concentrations, there was no significant difference in the Th17 ratios. The possible reasons for this lack of a difference in the Th17 frequency include limited sample size and the migration of Th17 cells. The first part of our study involved 46 study subjects, and for the second part of our study, we initially followed up with all of our study subjects. However, some of our subjects had already begun to receive treatment after their diagnosis, and such treatment would have confounded our results. Thus, for the second part of our study, we did not pursue patients who had already begun treatment. We also lost additional subjects to follow-up as a result of their unwillingness to participate further in our study. Moreover, the complexity and sensitive nature of flow cytometry makes the already small ratio of Th17 cells difficult to measure accurately.

We have shown that adults with OSAHS are more likely to exhibit an increase in circulating Th17 cells along with elevated IL-17A levels. Th17 cells are able to promote the activation, proliferation, and effector functions of a wide range of immune system cells, in contrast to Treg cells. Anderson and Tan recently described significant negative correlations between the severity of paediatric OSA and the percentage of Treg cells. An increase in the frequency of Th17 cells, together with a reduction in the frequency of Treg cells, could be a possible explanation for the link between OSAHS and inflammation [6,22].

Some authors have reported that daytime PAH was observed in severe OSAHS patients in the absence of significant heart or lung disease [23,24]. Moreover, OSAHS is frequently regarded as an independent risk factor for the development of pulmonary hypertension, and the European Respiratory Society (ERS) categorises OSAHS as a risk factor for PAH [25]. The ERS hypothesises that one crucial factor that may cause PAH in OSAHS is pulmonary vasoconstriction, which is a direct response to alveolar hypoxia and a cascade of biochemical and molecular events that culminate in pulmonary vascular remodelling [26,27]. However, PAH was not observed in our study, and we did not find any link between the severity of OSAHS and the level of PAP. The main reasons for this difference between the results of those studies and our results may be the limited number of patients studied, the length of illness, inter-individual differences in the response to factors such as hypoxia and different measurement methods for PAP.

There were also several limitations to our measurements of patients’ PAP. First, we used transthoracic echocardiographic indices to calculate PAP. Right heart catheterisation is the gold standard for the diagnosis of PAH [25]. However, the establishment of a definite diagnosis with this invasive method is always hampered by several factors, including the risk to patients and patient unwillingness to undergo to this procedure. Second, as a result of loss to follow-up, only 17 patients had their PAP measured.

## Conclusions

Our data offered direct evidence that IL-17A is elevated in patients with OSAHS and that the magnitude of this increase is associated significantly with the severity of OSAHS. As a pro-inflammatory cytokine, IL-17A may be an important factor linking OSAHS to systemic inflammation. However, the clinical utility of the IL-17A assay for the diagnosis of OSAHS and for the accurate evaluation of severity still needs to be studied further. In addition, large-scale, prospective studies are necessary to obtain better knowledge of the relationship among Th17 cells, PAP, and OSAHS.

## Abbreviations

AHI: Apnoea hypopnoea index; BMI: Body mass index; CRP: C-reactive protein; ELISA: Enzyme-linked immunosorbent assay; FITC: Fluorescein isothiocyanate; JAK: Janus kinase; LSaO2: Lowest arterial oxygen saturation; MSaO2: Mean arterial oxygen saturation; OSAHS: Obstructive sleep apnoea/hypopnoea syndrome; PAH: Pulmonary arterial hypertension; PAP: Pulmonary arterial pressure; PBMC: Peripheral blood mononuclear cell; PE: Phycoerythrin; ROR: Retinoid-related orphan receptor; ROS: Reactive oxygen species; STAT: Signal transducers and activators of transcription.

## Competing interests

The authors have no competing financial interests.

## Authors’ contributions

LY: Performed experiments, analyzed data, wrote manuscript; HQL: performed analyses and assisted in procuring human blood sample, reviewed manuscript; ZJP: carried out full-night polysomnography, collected data; SNM and PZ: aided in data collection and analyses;QW and GHL: performed ELISA and flow cytometry, produced graphs and tables; JYZ: designed the study and assisted in generating the final manuscript. All authors have read and approved the final manuscript.

## Authors’ information

Department of Respiratory Diseases,First Affiliated Hospital of Zhejiang University School of Medicine,79 Qingchun Road, Hangzhou, Zhejiang 310003, P.R. China

## Pre-publication history

The pre-publication history for this paper can be accessed here:

http://www.biomedcentral.com/1471-2466/14/84/prepub
